# The Impact of Gastrointestinal Hormones on Human Adipose Tissue Function

**DOI:** 10.3390/nu16193245

**Published:** 2024-09-25

**Authors:** Marcelina Radziszewska, Lucyna Ostrowska, Joanna Smarkusz-Zarzecka

**Affiliations:** Department of Dietetics and Clinical Nutrition, Medical University of Bialystok, ul. Mieszka I 4B, 15-054 Bialystok, Poland; lucyna.ostrowska@umb.edu.pl (L.O.); joanna.smarkusz-zarzecka@umb.edu.pl (J.S.-Z.)

**Keywords:** obesity, gastrointestinal hormones, hormones, adipose tissue, ghrelin, GIP, GLP-1, PYY, CCK

## Abstract

Background: Obesity is a global issue, the development of which depends on many interacting factors. Among these, hormones secreted in the gastrointestinal tract play an important role. The aim of this review was to assess the impact of these hormones on the functions of adipose tissue. Methods: The analysis was based on the latest research concerning both adipose tissue and gastrointestinal hormones. Results: It was found that these hormones can significantly affect adipose tissue, both directly and indirectly. Some hormones, when secreted in excess, can stimulate adipose tissue formation processes, while others can inhibit them. The impact of hormones depends on the location and type of adipose tissue as well as the physiological state of the body. It should also be noted that no hormone acts in isolation but in close cooperation with other factors. Conclusions: The relationship between gastrointestinal hormones and adipose tissue, and their role in obesity, is a complex and evolving field of study. Further research is necessary, particularly into the interactions between hormones and other factors, as well as their mutual interactions.

## 1. Introduction

Obesity is a chronic and relapsing condition characterized by excessive accumulation of adipose tissue. Global organizations suggest that obesity is a worldwide problem. According to the Organization for Economic Co-operation and Development (OECD) data from 2019, obesity and its health consequences will contribute to a reduction in life expectancy in OECD, Group 20 (G20) and European Union (EU28) member countries by 0.9−4.2 years in the years 2010–2030. Furthermore, it will cause approximately 92 million premature deaths in these countries [1]. A report released in 2020 by the World Obesity Federation states that by 2025, obesity will affect 21% of women and 18% of men. It is also projected that by 2030, this condition will affect over 1 billion people worldwide [2].

Obesity is often believed to be the result of an imbalance between energy intake and energy expenditure. However, it is important to note that the development of this condition is far more complex and depends on immunological, genetic, epigenetic, psychosocial, environmental, behavioral and cultural factors, and their interactions. The influence of the gut microbiome is also significant [3]. Obesity is often compounded by metabolic disorders such as type 2 diabetes, cardiovascular disease, non-alcoholic fatty liver disease and cancers [4]. 

A common problem in obesity is dysregulated energy homeostasis, which can lead to severe metabolic complications [3]. Metabolic disturbances are influenced by factors such as the location and type of adipose tissue, and the adipokines it produces [4]. There are three types of adipose tissue in the human body: white adipose tissue (WAT), beige adipose tissue (BeAT) and brown adipose tissue (BAT). Different types of adipose tissue have their own distinct structures, which are crucial for their respective functions. The primary function of WAT is the storage of energy derived from lipid metabolism. Therefore, it is predominantly composed of large adipocytes that harbor a single, large lipid droplet and a relatively small number of mitochondria. In contrast, BAT is characterized by a high number of mitochondria and small lipid droplets as it is primarily responsible for heat production [4,5]. BeAT is located within white adipose tissue since it is formed by WAT adipocytes that have acquired some properties of BAT [6]. WAT is located in two main depots: visceral white adipose tissue (VAT), which includes mesenteric, omental, gonadal, retroperitoneal and epicardial WAT, and subcutaneous white adipose tissue (SAT), located beneath the skin [5]. VAT is the most metabolically active and plays a significant role in the development of complications associated with obesity, such as certain cancers, non-alcoholic fatty liver disease, cardiovascular disease, lipid disorders and carbohydrate metabolism disorders. The impact of VAT on the development of metabolic disorders is associated with an increased production of pro-tumorigenic factors, elevated levels of pro-inflammatory cytokines, particularly IL-6 and C-reactive protein, and triacylglycerols [7,8]. It also contributes to decreased tissue sensitivity to insulin and reduced glucose uptake. Excessive accumulation of VAT is associated with vascular endothelial remodeling and dysfunction [4]. SAT, particularly located in the gluteal–femoral region, is less metabolically active than VAT and does not affect the development of metabolic diseases [6]. In contrast, BAT can counteract the negative effects of obesity and protect against hypertension, glucose and triglyceride imbalances in serum, and other components of the metabolic syndrome [4]. As noted, SAT can serve a protective function by long-term storage of fatty acids, which prevents the accumulation of visceral fat. Unlike VAT, SAT exhibits lower activity in the lipolysis process. However, BAT is associated with a beneficial adipokine profile; increased concentrations of leptin and adiponectin, and decreased levels of pro-inflammatory cytokines have been reported [9]. 

The functioning of adipose tissue is regulated by signals sent from the central nervous system as well as from other tissues and organs of the body [10]. The central nervous system regulates adipose tissue through the sympathetic nervous system by releasing catecholamines and norepinephrine [11]. The nervous system plays a crucial role in lipolysis, the browning of white adipocytes and thermogenesis, particularly in brown adipose tissue. It may also participate in the regulation of adipose tissue mass and its adaptive capacity as well as in the regulation of inflammatory processes within adipose tissue [11]. 

The functions of adipose tissue can also be regulated by hormones secreted in the body. Hormones are substances primarily secreted by endocrine glands. However, it has been observed that they can also be secreted by non-glandular cells located in various parts of the body, including the gastrointestinal tract [12]. Insulin is one of the hormones secreted by the pancreas that affects the function of adipose tissue by influencing the central nervous system. This hormone, upon binding to receptors in the brain, contributes to the inhibition of sympathetic nervous system activity and the suppression of lipolysis [11]. Insulin also acts directly on the metabolism of adipose tissue; it is essential in maintaining the structure of adipocytes and their differentiation. Upon binding to receptors within adipose tissue, it stimulates the accumulation of lipids in adipose tissue by promoting glucose uptake and lipogenesis. Insulin is also a powerful inhibitor of lipolysis [10]. 

In contrast to insulin, the process of lipolysis can be stimulated by hormones such as leptin, glucagon, cortisol, adrenocorticotropic hormone, thyroid and parathyroid hormones and growth hormone [10]. Hormones secreted in the body are also responsible for the redistribution of adipose tissue. It has been observed that in Cushing’s disease, characterized by increased secretion of glucocorticosteroids, these changes contribute to weight gain and stimulate the growth of visceral fat at the expense of the loss of limb fat. The significant impact of hormones can also be seen when comparing the distribution of adipose tissue in men and women. Oestrogens and androgens are responsible for the gynoid and android distribution of adipose tissue in women and men, respectively [10]. Thyroid hormones and their derivatives are also essential for the functioning of adipose tissue since they are responsible for the activation and differentiation of adipocytes. In BAT, they are crucial for thermogenesis, while in WAT, they initiate the browning process of white adipocytes [13]. 

It is also worth focusing on the hormones produced in the gastrointestinal tract as they play an important role in regulating the function of adipose tissue. These hormones regulate the sensations of hunger and satiety as well as the body’s energy metabolism. Human gastrointestinal hormones are secreted by endocrine cells, paracrine cells and neurons. Endocrine cells are distributed in the mucosa of the gastrointestinal tract among non-endocrine cells. These cells do not form gland-like structures. However, approximately 30 hormone genes are expressed in the intestines, making it the largest endocrine organ in the body. Hormones are secreted along the entire length of the gastrointestinal tract, from the stomach through the small intestine to the distal part of the colon [12]. Considering the impact of gastrointestinal hormones on the development of obesity and their influence on adipose tissue function, the aim of this study was to characterize the mechanisms by which gastrointestinal hormones affect adipose tissue. 

## 2. Materials and Methods

A systematic literature search was conducted in the PubMed database to identify studies relevant to the current review. The following search strategy was employed: “obesity” OR “fat tissue” OR “WAT” OR “BAT” OR “SAT” OR “VAT” OR “white adipose tissue” OR “brown adipose tissue” OR “visceral adipose tissue” OR “subcutaneous adipose tissue” OR “hormones” OR “gastrointestinal hormones” OR “ghrelin” OR “GIP” OR “glucose-dependent insulinotropic peptide” OR “GLP-1” OR “glucagon-like peptide-1” OR “PYY” OR “peptide YY” OR “CCK” OR “cholecystokinin”) and (“homeostasis” OR “functions” OR “metabolism” OR “physiology” OR “secretion” OR “morphology” OR “fat tissue” OR “white adipose tissue” OR “obesity”). We aimed to limit the search to articles from the past five years; however, in the absence of relevant data, studies from earlier years were also included. Out of 15,000 references found, 54 meta-analyses, reviews and clinical studies were selected for the preparation of the article. Only studies published in English, to which we had full access, were included. The following inclusion criteria were applied: studies lasting longer than one day, involving both an experimental group and a control group, and conducted exclusively with adult participants. In the absence of human studies, animal studies were considered. Studies that used blood tests, adipose tissue biopsies, various body composition measurement methods and methods for assessing energy expenditure were analyzed in the present review. Exclusion criteria included lack of access to full-text articles, imprecise results and study designs, studies lasting less than one day, absence of both experimental and control groups, involvement of children and limited relevance to the topic.

## 3. Results

Hormones secreted in the gastrointestinal tract are primarily known for their effect on the sensation of hunger and satiety. Therefore, they are categorized into orexigenic hormones, which stimulate the hunger center, and anorexigenic hormones, which suppress appetite and promote the feeling of fullness. Ghrelin is the main orexigenic hormone, the levels of which reach a peak during a 24−48 h fast and prior to meal consumption. During meal consumption, nutrients come into contact with gastrointestinal cells responsible for producing anorexigenic hormones, resulting in the release of, among others, glucagon-like peptide-1 (GLP-1), peptide YY (PYY) and cholecystokinin (CCK). Stimulation of the central nervous system by the released hormones results in the sensation of satiety [14,15]. However, gastrointestinal hormones can also perform other functions in the body and, as a result, have a significant impact on maintaining overall homeostasis. These hormones affect, among other things, the cardiovascular system, glucose and insulin metabolism, liver function, muscle function and bone mineralization. They also have a substantial impact on lipid metabolism and adipose tissue function. Therefore, in this review, we aim to focus on the effects of gastrointestinal hormones on adipose tissue, particularly in individuals with obesity.

### 3.1. Ghrelin

Ghrelin is a peptide consisting of 28 amino acids. It was first described in 1999. At that time, its secretion was discovered and research into its functions in the human body began [16]. Ghrelin is primarily secreted by cells located in the submucosal layer of the gastric fundus. Additionally, it is released in smaller amounts by cells in the gastric body, duodenum, jejunum, placenta, kidneys, heart and thyroid gland. The hormone can be secreted in one of two forms: the acylated form, which accounts for approximately 10% of the secreted ghrelin, and the unacylated form, which constitutes 90% of the released hormone [17]. Unacylated ghrelin is converted into its active form, acylated ghrelin, through the enzyme ghrelin O-acyltransferase (GOAT) [18]. The secretion of ghrelin can be regulated by various factors, including physical activity, lifestyle and the environment [17]. Ghrelin serum concentration is strongly dependent on variations in nutritional status, meal intake and the proportions of nutrients in the meal [19,20,21,22]. Fasting leads to increased ghrelin production in the stomach and elevated ghrelin concentrations in the blood [23]. The first function of ghrelin discovered by researchers was its strong stimulation of growth hormone secretion [16]. Ghrelin is often referred to as the “hunger hormone” because it plays a key role in stimulating the sensation of hunger and increasing food intake as well as limiting energy expenditure and promoting fat storage. Additional actions of ghrelin in the human body include stimulating gastric motility and gastric emptying, influencing the proliferation and apoptosis of pancreatic cells, enhancing taste perception, affecting the metabolism of adipose tissue and glucose, regulating cardiovascular function, protecting against muscle mass reduction, influencing bone metabolism and regulating sleep [24]. 

Ghrelin can affect adipose tissue both through the central nervous system and by direct action on adipocytes. A study by Theander-Carrillo et al. evaluated whether central administration of ghrelin influenced peripheral metabolism and whether this effect was dependent on its orexigenic action [25]. The study was conducted on rats which were divided into four groups. It was observed that in the group of animals that were administered ghrelin and were allowed unrestricted access to food, weight gain was significantly greater compared to the other groups. The weight gain among the rats receiving ghrelin was associated with an increase in fat mass without significant changes in lean body mass. Using indirect calorimetry, it was demonstrated that ghrelin administration did not contribute to changes in total energy expenditure or energy expenditure induced by spontaneous physical activity. However, a significant increase in the respiratory quotient was observed, indicating increased lipid storage. The increased clearance of triacylglycerols (TGs) among the rats receiving ghrelin reflected lower serum TG levels compared to the control groups. The study showed that ghrelin infusion significantly increased the expression of genes encoding enzymes responsible for lipid metabolism and storage, such as lipoprotein lipase, fatty acid synthase, acetyl-CoA carboxylase and stearoyl-CoA desaturase-1, while decreasing the synthesis of the enzyme responsible for fat oxidation. This mechanism contributed to increased glucose uptake in both WAT and BAT. However, in BAT, the hormone decreased UCP expression, leading to reduced heat production. Ghrelin’s impact on adipose tissue is independent of its orexigenic effect as metabolic changes within adipocytes were observed in both experimental groups administered ghrelin. The authors suggest that central administration of ghrelin, through its action on the sympathetic nervous system, contributes to the regulation of energy metabolism within adipose tissue. They also indicate that further research may provide an opportunity to develop ghrelin receptor antagonists or agonists with a therapeutic potential [25]. 

The impact of ghrelin on lipid metabolism in white adipose tissue has also been confirmed in studies by Sangiao-Alvarellos et al. [26]. The authors used rat models, with wild-type rats as the control group and growth hormone-deficient rats as the experimental group, to investigate how central administration of ghrelin affects lipid metabolism in adipose tissue and the liver. The results indicate that ghrelin increases the activity of enzymes responsible for lipid synthesis in both WAT and the liver, independent of growth hormone, while simultaneously inhibiting the enzymes responsible for lipid oxidation. However, it was observed that the inhibitory effect on adipocytes is independent of growth hormone secretion while the effect in the liver is dependent on growth hormone. The authors conclude that the potential for fat storage in the liver is higher when growth hormone is also involved since this promotes both lipid accumulation and reduced lipid uptake. In contrast, lipid storage in WAT is effective regardless of growth hormone secretion. The authors indicate that further studies focusing on understanding the mechanisms of ghrelin’s action on WAT could be used to develop drugs for the treatment of obesity and related comorbidities [26]. 

The effects of ghrelin infusion were also evaluated in a study by Davies et al. [27]. The authors used rat models to assess how intravenous infusion of acylated ghrelin affected abdominal adipose tissue. It was observed that after a two-week continuous infusion of acylated ghrelin, there was an increase in VAT volume with no changes in SAT. Furthermore, a one-week infusion of unacylated ghrelin did not lead to changes in adipose tissue. Following the evaluation of glucose and lipid levels in serum, it was found that ghrelin does not contribute to an increase in abdominal obesity through the enhanced availability of substrates. However, it promotes the development of abdominal obesity by reducing lipid mobilization from adipose tissue, as evidenced by the assessment of enzyme activity changes which showed decreased activity of enzymes responsible for lipid oxidation. It is also noteworthy that acylated ghrelin infusion contributed to liver steatosis by stimulating triacylglycerol storage. The authors conclude that discontinuing ghrelin delivery to adipose tissue may be effective in reducing adipose tissue mass, particularly in the treatment of metabolic syndrome [27].

Two reviews of studies investigating the effects of ghrelin on adipose tissue content have been conducted and it has been observed that ghrelin decreases the utilization of lipids as an energy source, resulting in increased fat storage and weight gain [23,28]. Based on the reviewed studies, Abizaid et al. identified four mechanisms through which ghrelin influences the development of obesity. The hormone contributes to metabolic changes that increase the utilization of carbohydrates as an energy source, resulting in the accumulation of lipids in adipose tissue. Additionally, ghrelin enhances the preference for fat consumption, leading to increased energy intake. Another effect of ghrelin on obesity development is the reduction in basal metabolic rate and thermogenesis. The final mechanism observed by the authors focuses on decreased spontaneous physical activity due to ghrelin’s action, resulting in reduced energy expenditure. However, the authors of the study highlight the need for further research to explore the precise mechanisms by which ghrelin affects body homeostasis and to develop new approaches for treating obesity [28]. 

In summary, ghrelin is a hormone that plays an important role not only in stimulating appetite and food intake, but also in lipid metabolism within adipose tissue. The mechanisms by which ghrelin affects adipose tissue should be thoroughly investigated but it is clear that it contributes to increased lipid storage in adipocytes and thereby promotes weight gain. A detailed understanding of ghrelin’s impact on adipose tissue may lead to the development of new therapeutic interventions for weight reduction.

### 3.2. Gastric Inhibitory Polypeptide (GIP)

Gastric inhibitory polypeptide (GIP), a 42-amino-acid incretin hormone, exerts effects opposite to those of ghrelin [29]. The hormone is secreted by K cells, primarily located in the duodenum and the proximal segments of the small intestine [30]. Its secretion is stimulated by food intake, with a particularly rapid release occurring a few minutes after the consumption of glucose or other easily absorbed carbohydrates, including sucrose. The stimulation of GIP secretion regulates carbohydrate metabolism by promoting insulin release. Although fats and proteins also stimulate GIP release, they do not trigger insulin secretion [30,31]. GIP concentration increases most rapidly and most markedly within 30 min of the start of a meal, and a consistently high level is maintained for three hours after meal ingestion. Hormone secretion is proportionally dependent on the size of the meal. A study by Alsalim et al. demonstrated that administering a higher-calorie meal, but with the same nutrient composition, leads to increased GIP secretion [32]. Additionally, GIP secretion is dependent on the timing of meal consumption as demonstrated in a study by Lindgren et al. It was observed that the secretion of this incretin is higher after meal ingestion in the morning compared to meal ingestion in the afternoon. The authors conclude that this may contribute to the more rapid early insulin response, stronger stimulation of pancreatic beta cells and more rapid reduction in serum glucose levels after the morning meal [33]. However, GIP secretion is independent of the rate of meal ingestion [34]. The first function of GIP discovered by researchers was inhibition of gastric acid secretion [30]. The main function of this hormone is the stimulation of insulin secretion, which occurs through the activation of pancreatic beta cells. However, it is important to note that the incretin effect of GIP is activated only in the presence of high blood glucose levels [31]. Other functions of GIP include reducing food intake by stimulating the satiety center, anti-atherogenic effects and promoting bone formation [35,36]. Although its insulinotropic effects have been elucidated in previous studies, the impact of GIP on adipose tissue remains unclear [36].

In 2018, an animal study evaluating the use of GIP receptor antagonists in the treatment of obesity was conducted [37]. The study examined obese animals. The animals were divided into two groups and the authors measured their biochemical and physiological markers, conducted histochemical examinations and performed gene expression analysis. A significant reduction in body weight was observed among the animals receiving GIP receptor antagonists. However, further research is needed to elucidate the mechanisms behind the observed weight loss. The authors also found that GIP receptor antagonists increase insulin sensitivity and glucose utilization, which results in reduced accumulation of fatty acids in the liver and skeletal muscles. Enhanced tissue insulin sensitivity may also have a positive impact on diabetes progression. The authors of the study suggest that GIP receptor antagonists, through their positive effects on lipid and glucose metabolism, may be beneficial in the treatment of diabetes and obesity [37]. 

In a study by Sakane et al., the effects of a specific diet on GIP secretions and visceral fat area were examined [38]. The study involved men with obesity or overweight without type 2 diabetes who followed a specific dietary regimen for a period of two weeks. It was observed that among individuals consuming a traditional Japanese diet (characterized by limited consumption of animal products, meat and animal fats, with a high intake of fish and soy products), there was a decrease in the levels of LDL cholesterol and glycated hemoglobin, and improvement in the HOMA-IR index. This diet is low in fat and the authors suggest that lower fat intake contributes to the reduced release and lower serum GIP levels. Additionally, a reduction in VAT was noted in the study group, which was associated with decreased serum GIP levels. The authors observed that individuals with obesity exhibit reduced GIP activity in SAT, but not in VAT. It was therefore suggested that GIP contributes to the development of obesity by promoting lipid accumulation in VAT. The authors conclude that lowering GIP levels through diet may improve metabolic outcomes and reduce body weight. However, further studies are necessary to elucidate the exact mechanisms regulating the impact of GIP on the body [38]. 

A review by Seino et al. indicates that GIP may contribute to the development of obesity, particularly among individuals consuming a high-fat diet. Current dietary patterns are rich in high-fat dairy products, red meat and animal fats. Studies show that contemporary eating habits lead to increased lipid accumulation in adipose tissue due to enhanced GIP secretion and the expression of GIP receptors in adipose tissue [39]. However, the authors emphasize the need for further studies to understand the precise mechanisms of GIP action in obesity.

A recent review by Kagdi et al. analyzed studies that used cell cultures, animal models or humans to determine the impact of GIP on adipose tissue metabolism [40]. Furthermore, the authors attempted to assess whether agonism or antagonism of the GIP receptor positively affected adipose tissue function. It was observed that the GIP receptor is present throughout adipose tissue, including both white and brown adipose tissue. Existing studies demonstrate that GIP receptors in adipose tissue are involved in reducing adipose tissue mass, lipolysis, re-esterification of free fatty acids, glucose and lipid uptake and removal, lipid oxidation and blood flow through adipose tissue. Both GIP receptor agonists and antagonists may positively influence adipose tissue function, and their effects may be dependent on other hormones such as insulin and GLP-1. The authors suggest that drugs targeting the GIP receptor could be useful in treating metabolic disorders, type 2 diabetes and obesity. Studies conducted on cell cultures, animals and humans have shown that GIP receptor co-agonists or triagonists impact body weight reduction and improve lipid profiles. However, further research is needed to evaluate the exact mechanisms of their action and interactions with other hormones. It is also crucial to consider the location of adipose tissue, as this hormone exhibits different effects depending on the location of adipose tissue, which has not yet been fully explored and understood [40].

A study by Regmi et al. evaluated the impact of tirzepatide on nutrient metabolism and the function of the GIP receptor in adipose tissue [41]. Tirzepatide is a novel dual GIP and GLP-1 receptor agonist. Clinical studies conducted to date have demonstrated that it is more effective in reducing body weight and serum glucose and triglyceride levels compared to a selective GLP-1 receptor agonist. However, none of the studies have elucidated the precise mechanisms of action of GIP receptor agonists. The study used human adipocytes and murine models to determine the impact of GIP receptor agonists on adipose tissue cell function during fasting and in the postprandial state. Functional assays revealed that GIP receptor agonists improve insulin signaling, glucose uptake and glucose conversion to glycerol. It was also shown that in the absence of insulin, GIP receptor agonists enhance the intensity of lipolysis. High-fat-diet-induced mice that were treated with long-acting GIP receptor agonists demonstrated reduced circulating triglyceride levels during oral lipid loading and increased uptake of fatty acids from lipoproteins into adipose tissue. These findings support a model of action for long-acting GIP receptor agonists which differentially regulate adipose tissue function both in fasting and in the postprandial state by cooperating with insulin to enhance glucose and lipid clearance after meals while increasing lipid mobilization during fasting states, when reduced insulin levels are present [41].

In summary, the current body of research on the effects of GIP on adipose tissue is insufficient to accurately assess the impact of the hormone on adipocytes. The mechanisms of GIP action remain unclear. The majority of existing studies suggest that GIP may contribute to increased body weight through the accumulation of adipose tissue, particularly VAT. Some studies even indicate the beneficial use of GIP antagonists in weight reduction. However, recent research highlights the potential advantages of long-acting GIP receptor agonists in influencing lipid and glucose metabolism within adipocytes. Therefore, further research is necessary to clarify how GIP interacts with adipose tissue, with particular attention to the differentiation between SAT and VAT. 

### 3.3. Glucagon-like Peptide-1 (GLP-1) 

Glucagon-like peptide-1 (GLP-1) is the second incretin hormone besides GIP. This hormone exists in two forms, GLP-1 (7-36) and GLP-1 (7-37), although the GLP-1 (7-37) form is produced in larger quantities. It has been observed that GLP-1 is mainly secreted in three areas of the human body. The largest amounts are released by enteroendocrine L-cells located within the intestines [42]. It has been demonstrated that L-cell density within the duodenum and the proximal part of the small intestine is low, but increases in the distal parts of the intestines, with the highest concentration observed in the ileum and the colon [43]. This hormone is also secreted in the pancreas by alpha cells and in the central nervous system [42]. GLP-1 secretion is primarily stimulated by nutrients in ingested food. It has been observed that the infusion of glucose or lipids into the ileum causes immediate GLP-1 release [44]. However, during the consumption of a meal, GLP-1 secretion is delayed due to the time it takes the nutrients to reach the area with the highest concentration of L-cells [45]. The location of L-cells and the delayed release of GLP-1 explain the prolonged high concentration of GLP-1 in the blood serum during a meal, particularly among individuals who have undergone gastric bypass surgery or sleeve gastrectomy [43]. It is worth noting that the amount of GLP-1 secreted depends on the composition and size of the meal. A meal with a higher energy content contributes to a more significant increase in systemic total and active GLP-1 levels compared to a meal with a lower energy content [46]. The most potent nutrients stimulating GLP-1 secretion are monosaccharides such as glucose, fructose and galactose. It has been observed that the increase in hormone levels occurs more rapidly after the consumption of proteins and carbohydrates. After the consumption of fats, on the other hand, the rise in GLP-1 levels happens later, but the high concentration persists for a longer duration [43]. It is important to note that postprandial GLP-1 levels depend on the site of sample collection. It has been demonstrated that 75% of active GLP-1 is degraded in the gastrointestinal tract, and after reaching the liver, an additional 50% of the hormone is broken down, with the remaining portion entering systemic circulation. It is estimated that only 10–15% of active GLP-1 reaches the pancreas [43,47]. Research on GLP-1 focuses mainly on its role in stimulating insulin secretion by activating pancreatic beta cells and the potential use of hormone analogs for the treatment of type 2 diabetes and obesity. Beyond its incretin effects, GLP-1 suppresses appetite and food intake, delays gastric emptying, and exhibits anti-inflammatory, anti-atherosclerotic, cardioprotective, antihypertensive and neuroprotective actions. It also protects the peripheral nervous system, alleviates dyslipidemia, and provides renal protection in diabetic nephropathy and retinal protection in diabetic retinopathy [35]. GLP-1 may contribute to weight reduction both by suppressing appetite and regulating glucose metabolism, but it is also suspected that it directly affects changes in adipose tissue.

A study by Kadouh et al. assessed the impact of liraglutide, a GLP-1 analog, on body weight and body composition [48]. The study involved individuals with overweight (BMI ≥ 27 kg/m^2^) or obesity (BMI > 30 kg/m^2^). The results demonstrated statistically significant differences in total body weight between the groups. In the liraglutide group, body weight reduction was approximately 5.8 kg, while in the control group, it was around 1 kg. It was also observed that fat tissue in both the lower and upper body regions, as well as visceral fat tissue, was significantly reduced in the liraglutide group compared to the placebo group. Notably, no changes in lean body mass were observed in the study. The authors noted that the weight loss effect was attributed to the delayed gastric emptying induced by the GLP-1 analog and significantly reduced appetite. Importantly, the weight loss was associated with a reduction in fat tissue without changes in lean body mass, suggesting that GLP-1 may directly affect adipose tissue. However, the mechanisms by which GLP-1 acts on adipose tissue remain unidentified [48]. 

It has been observed that GLP-1 receptors are present in adipose tissue, and therefore, it has been suggested that this hormone is involved in lipid metabolism. The impact of liraglutide on the composition and function of adipose tissue was analyzed by Wegeberg et al. [49]. The study included individuals with type 2 diabetes and polyneuropathy. To assess the composition and function of adipocytes, a biopsy of adipose tissue was obtained from each study participant. It was hypothesized that GLP-1 might affect adipose tissue morphology through its impact on the autonomic nervous system, and by influencing the balance between lipogenesis and lipolysis via GLP-1 receptors located in adipose tissue. The findings indicate that liraglutide leads to weight reduction but does not affect the size of subcutaneous adipocytes, pericellular fibrosis or CD163-positive macrophage infiltration. Furthermore, the study demonstrated that the GLP-1 analog did not contribute to changes in the serum levels of free fatty acids, CD163, leptin or adiponectin, which could reflect changes in subcutaneous fat composition. The authors suggest that these results may explain a more significant impact of GLP-1 on the reduction in visceral fat tissue. However, they pointed out the need for more detailed studies to evaluate the effects of GLP-1 on adipose tissue composition and function [49].

A meta-analysis by Liao et al. in 2022 examined studies evaluating the effects of GLP-1 receptor agonists on visceral fat tissue composition and hepatic fat content [50]. The results of the analyzed studies show that the use of GLP-1 analogs significantly reduces visceral fat tissue and hepatic fat content compared to lifestyle interventions, placebo and other medications. GLP-1 analogs effectively reduced hepatic fat tissue in both healthy individuals and those with type 2 diabetes and non-alcoholic fatty liver disease. The authors demonstrated that GLP-1 affects fat reduction by acting on the central nervous system to decrease appetite as well as by directly binding to receptors in adipose tissue, thereby limiting the formation of WAT and hepatic fat. However, the precise mechanisms of the impact of GLP-1 on adipose tissue have not been fully described [50].

It is worth noting that both GIP and GLP-1 circulate in very low concentrations. For this reason, a very important final step in both clinical and preclinical studies is the assessment of concentrations with very sensitive assays, which should be included in any ongoing study assessing the function of these hormones [47].

In summary, it should be noted that there is a limited body of research on the impact of GLP-1 on adipose tissue. The majority of the existing studies indicate that GLP-1 contributes to weight reduction primarily by decreasing appetite. However, it is known that GLP-1 receptors are also present in adipose tissue. Furthermore, the administration of GLP-1 analogs leads to a reduction in fat tissue, suggesting that GLP-1 must have an effect on the composition and function of adipose tissue. GLP-1 exerts a particularly beneficial effect on the reduction in visceral fat, which is significant in the treatment of various metabolic disorders, including obesity, type 2 diabetes and non-alcoholic fatty liver disease. Nevertheless, the mechanisms of this action are not yet fully understood. Therefore, there is a need for more human studies to assess the mechanisms of GLP-1 action in adipose tissue. New research may expand the knowledge of GLP-1 and may contribute to the development of new applications for GLP-1 analogs in the treatment of various metabolic diseases.

### 3.4. Peptide YY (PYY) 

L-cells of the gastrointestinal tract also secrete peptide YY (PYY), besides releasing GLP-1. PYY is a hormone composed of 36 amino acids, with a tyrosine residue at each end [51,52]. PYY has a very similar structure to pancreatic polypeptide and neuropeptide Y [51]. In the bloodstream, PYY exists as PYY (1-36) and PYY (3-36), the latter of which is formed through the proteolysis of PYY (1-36) by the enzyme dipeptidyl peptidase IV [53]. PYY concentration increases postprandially, particularly following the consumption of a high-fat meal [54]. Postprandial PYY concentration is proportional to the amount of energy consumed [51]. Similarly to GLP-1, PYY reduces appetite by acting on the central nervous system and inhibiting neuropeptide Y activity through binding to the Y2 receptor [55]. It stimulates the feeling of satiety, thereby contributing to a reduction in the amount of food intake. It also delays gastric emptying by limiting gastric peristalsis and decreasing the secretion of pancreatic hormones. It has been demonstrated that PYY is integral to maintaining the body’s energy homeostasis. However, its role in the development of obesity remains controversial and unclear, as the mechanisms of PYY action have been understudied and underanalyzed [52].

The poorly understood effects of PYY on other metabolic processes, beyond appetite suppression, were the subject of research by Adams et al. [54]. In the study, mice were administered subcutaneous infusions of PYY (the experimental group) or placebo (the control group). It was observed that the experimental group experienced significant weight loss and displayed reduced food intake. Body composition analysis revealed a substantial loss of fat tissue in the animals receiving PYY. It is worth noting that no differences in lean body mass were observed between the groups. The hormone infusion also led to a significant reduction in the respiratory quotient, which might reflect the effect of PYY on increased fatty acid oxidation. The authors of the study suggest that the anorectic effect of PYY is not the only mechanism of energy deficit leading to weight reduction. This hormone contributes to maintaining the metabolic rate characteristic of body weight under conditions of reduced food intake. Additionally, it results in a significant reduction in fat tissue in conjunction with increased lipid burning throughout the body [54]. A similar study was conducted by van den Hoek et al. [56]. The research team also used mice and administered either PYY infusions or placebo to assess the impact of the hormone on energy expenditure. The authors observed a reduction in the respiratory quotient in the PYY infusion group, indicating that the hormone decreases glucose utilization as an energy source while increasing lipid utilization. This mechanism may contribute to weight loss. It is worth noting that this effect is independent of the anorectic action of PYY. However, the precise mechanisms of PYY action and its impact on lipolysis have not been determined. It was also observed that PYY infusion increases glucose uptake in adipose tissue, which is also independent of its effect on appetite. These documented effects do not diminish with prolonged use, creating potential for the application of PYY in the treatment of metabolic syndrome. However, further research, particularly in humans, is necessary [56].

Abdel-Hamid et al. also evaluated the impact of PYY on adipose tissue [53]. Diabetic rat models were used in the study. The control group was administered saline or sodium citrate buffer as a placebo, while the experimental group received PYY infusions. It was observed that the PYY-treated group experienced a significant reduction in body weight and visceral fat content, and their food intake was decreased compared to the other groups. Furthermore, a decrease in mRNA expression of NF-kappaB in the adipose tissue of the experimental group was observed. The authors suggest several possible mechanisms by which PYY reduces visceral fat: increased utilization of lipids through a direct effect on adipose tissue, stimulation of Y2 receptors in visceral fat or reduction in the expression of neuropeptide Y in the central nervous system. By reducing visceral fat, PYY lowers NF-kappaB expression, which results in decreased IL-6 production and reduced systemic inflammation. The impact of PYY on inflammatory responses contributes to a lower risk of developing metabolic diseases associated with obesity. Therefore, the authors indicate that PYY could be a promising method for treating obesity and type 2 diabetes. However, there is a need for more research to precisely evaluate the mechanisms by which the hormone affects the structure and function of adipose tissue, particularly visceral fat [53]. 

In summary, while the effect of PYY on appetite regulation has been relatively well studied, its impact on other processes in the human body is not yet fully understood. Research has produced conflicting results regarding its influence on adipose tissue metabolism. Therefore, this hormone should undergo further clinical studies, particularly involving individuals with obesity, to determine whether PYY may affect fat content and, consequently, the development of obesity and its complications. More extensive research could create opportunities for developing new treatment methods for obesity. 

### 3.5. Cholecystokinin (CCK)

Cholecystokinin (CCK) also affects the feeling of satiety. It is a hormone secreted by endocrine cells in the duodenum. CCK secretion is stimulated by food intake, particularly fats and proteins. The most important function of CCK is to stimulate the pancreas to release digestive enzymes and to promote the contraction of the gallbladder. Additionally, it is involved in stimulating intestinal motility and insulin secretion by the pancreas. CCK enhances the sensation of fullness and, as a result, helps to reduce meal size and regulate food intake. It also decreases energy expenditure and delays gastric emptying. When stimulated by lipids, the released hormone plays a crucial role in the transport and metabolism of fats as well as in glucose homeostasis and maintaining energy balance between food intake and energy expenditure [57]. In collaboration with leptin and stimulating its release from adipocytes, CCK indirectly promotes fat catabolism through both peripheral and central mechanisms [58]. The activity of CCK is primarily dependent on its effects on specific CCK1 receptors which are located in the brainstem and peripheral afferent nerves. In the central nervous system and peripherally, CCK2 receptors play a crucial role in maintaining the normal structure of the gastric mucosa and may also influence lipid metabolism through the action of CCK [59]. 

A study conducted by Plaza et al. in 2018 aimed to assess the impact of CCK on lipid storage in WAT [58]. The authors attempted to confirm the hypothesis that CCK regulates the uptake of triacylglycerols by WAT through CCK receptors located in adipocytes. The study used rats that were administered saline (the control group), CCK or CCK antagonists. The study showed that CCK administration decreases the expression and circulating levels of angiopoietin-like protein 4 (ANGPTL-4), resulting in increased LPL activity in SAT and VAT. The in vitro study demonstrated that the action of CCK within adipocytes is dependent on CCK2 receptors. Importantly, the effect of CCK on adipose tissue was maintained during long-term observation. The authors concluded that the results indicate a significant physiological role of CCK in maintaining lipid and energy homeostasis. CCK may also complement the role of insulin in regulating triacylglycerol accumulation. Based on the findings, the authors believe that there is potential for using CCK agonists to regulate the function of WAT under conditions of insulin resistance. However, to develop new pharmacological treatments for insulin resistance, more studies are needed, particularly those involving human adipose tissue [58].

The impact of CCK on WAT was re-analyzed by Plaza et al. in 2019. The study evaluated the effect of CCK on the release of adiponectin by adipocytes [59]. Adiponectin is one of the most abundantly secreted and expressed adipokines. It plays a crucial role in lipid and glucose metabolism. Its plasma concentration is positively associated with increased insulin sensitivity. The action of adiponectin is particularly important in individuals with obesity, and its low concentration is often detected in people with insulin resistance and type 2 diabetes. It is currently considered an independent risk factor for insulin resistance, diabetes and cardiovascular disease. In the study, experiments were conducted both in vivo on rats and in vitro on isolated preadipocyte cells. The effects of CCK on adipocytes were obtained by using selective antagonists of the CCK1 and CCK2 receptors. One group of animals was administered saline, while the other received CCK. In the in vitro study, selective silencing of the CCK1 and CCK2 receptors was performed. Additionally, the influence of insulin on the action of CCK was determined. The study showed that CCK increased adiponectin production in adipocytes, particularly in SAT, but also in VAT adipocytes, leading to an increased plasma concentration of adiponectin. This effect was sustained over a long period. In the in vitro study, an increase in adiponectin secretion was also observed following the application of CCK. The action of CCK within adipose tissue, as evidenced by the results obtained in the study, is dependent on the CCK2 receptor. It is noteworthy that inactivation of the insulin receptor inhibited the stimulatory effect of CCK on adiponectin release in preadipocytes. In contrast, insulin administration enhanced the effect of CCK. Therefore, the authors concluded that CCK significantly stimulates the release of adiponectin via CCK2 receptors and that this effect is dependent on the presence of insulin. Due to its influence on adiponectin release, CCK was noted to have anti-inflammatory effects, as it tended to inhibit the release of pro-inflammatory cytokines such as IL-6, IL-1β and TNF-α in both SAT and VAT. The conclusions drawn from the analysis provide a basis for more extensive research, especially in human subjects, regarding new treatment possibilities for insulin resistance, particularly in individuals with obesity, in whom adiponectin production is reduced [59].

Following the two earlier studies, Plaza et al. focused on a third project in 2022 that investigated the role of CCK in adipose tissue [60]. The study evaluated the role of CCK in regulating the expression and function of the aquaglycerol channel aquaporin 7 (AQP7). AQP7 is a protein responsible for transporting glycerol across the adipocyte membrane into the bloodstream, which plays a crucial role in maintaining adipocyte homeostasis and insulin sensitivity. This action prevents the accumulation of glycerol within the cells, which occurs only in pathophysiological conditions and which negatively affects insulin sensitivity, thereby contributing to the development of obesity. To assess the effect of CCK on the secretion of this regulatory protein in SAT and VAT, in vivo studies on rats and in vitro studies on preadipocytes were conducted. The analysis also included the role of insulin receptors in the action of CCK on preadipocytes devoid of insulin receptors. The study design was similar to the previously described investigations.

The results demonstrated that CCK contributed to an increased expression of AQP7 in white adipose tissue of rats, leading to elevated blood glycerol levels. Similar results were obtained in the in vitro analyses. However, CCK did not affect the amount of fatty acids secreted by adipocytes, indicating that the effect of CCK was separate from the rate of lipolysis. Furthermore, it was noted that CCK did not enhance the phosphorylation of hormone-sensitive lipase, a step necessary for reducing the rate of lipolysis. Analysis of the influence of insulin and its receptors revealed that inactivation of the receptor halted CCK-dependent AQP7 expression in preadipocytes. It was also observed that insulin enhanced the action of CCK. The authors concluded that the synthesis and function of AQP7 are dependent on CCK, but this effect is closely tied to the influence of insulin. Therefore, appropriately selected CCK receptor agonists could have a positive impact on the maintenance and improvement of insulin sensitivity in WAT [60].

In summary, CCK positively affects the function of adipose tissue through various mechanisms. The results of the reviewed studies indicate that the use of CCK agonists may provide therapeutic effects in the treatment of insulin resistance. The pharmacological methods currently used to treat diabetes could be supported by the application of CCK agonists. However, to implement this new method in humans, it is essential to conduct a larger number of extensive clinical trials. These studies should also focus on separate analyses of VAT and SAT to determine where CCK causes greater changes and what the implications are. Currently, there is a limited amount of research on the effect of CCK on adipose tissue function, and the existing research has been conducted using exclusively animal models. Expanding the knowledge on how CCK affects tissue function in individuals with metabolic disorders could provide an opportunity for the development of new and more effective treatment methods.

However, it is important to note that no hormone in the human body acts independently. The proper functioning of each tissue requires the collaboration of multiple hormones. Therefore, future research should focus on analyzing the interactions of these hormones within the human body, with particular attention to their mutual effects on adipose tissue function.

**Figure 1:**
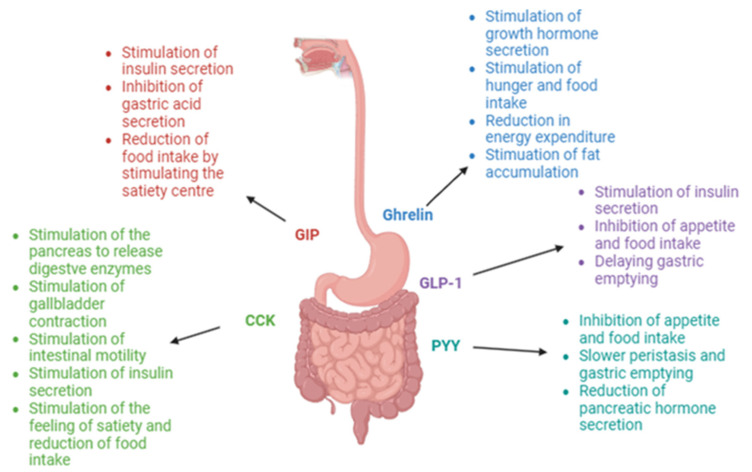
Main mechanisms of action of hunger/satiety hormones secreted in the gastrointestinal tract [16,24,30,31,35,36,52,55,57].

Table 1 presents the main results of all the reviewed studies. Figure 1 presents the main mechanisms of action of hunger/satiety hormones secreted in the gastrointestinal tract.

## 4. Physiological versus Pharmacological Effects of GIP and GLP-1

It is worth noting that systemic concentrations of GIP and GLP-1 are significantly lower than those observed pharmacologically, raising the possibility that physiology and pharmacology operate through different mechanisms. Studies conducted so far have shown that the endogenous GIP-GIPR system has a significant impact on the development of obesity [61]. As noted, individuals with obesity exhibit elevated fasting levels of GIP, as well as excessive GIP secretion following meal consumption. Studies conducted on animal models with diet-induced obesity have shown that these subjects display hyperplasia of K cells and increased GIP production. The most significant study demonstrating the role of GIP in the development of obesity was conducted by Miyawaki et al. [62]. In this study, it was observed that mice on a high-fat diet exhibited increased GIP production, along with significant accumulation of visceral and subcutaneous fat, as well as insulin resistance. However, mice lacking the GIP receptor, despite also being on a high-fat diet, were protected from both obesity and insulin resistance [36,62]. It has been suggested that inhibiting GIPR activity and endogenous GIP may be effective in treating obesity, particularly when it is diet-induced through a high-fat diet [61]. However, subsequent studies have shown that GIP analogs may also have a beneficial effect in the treatment of obesity [36,63]. A study conducted on animals by Mroz et al. demonstrated that a GIP analog, precisely tailored in terms of receptor selectivity, duration of action, and interspecies activity, is more effective in treating obesity than a GIP antagonist [63]. In a study conducted on animals by Zhang et al., it was observed that central administration of GIP analogs resulted in reduced body weight and food intake [36]. These observations indicate that the effects of GIP analogs primarily rely on their action in the central nervous system, leading to reduce food intake and increased insulin sensitivity. Additionally, the best outcomes were achieved when GIP analogs were combined with GLP-1 analogs [36,63]. However, in the observations by Zhang et al., it was also noted that GIPR antagonism increases blood flow to adipose tissue and nutrient delivery. The researchers concluded that GIP antagonists affect weight loss through peripheral mechanisms in adipose tissue, while GIP agonists act centrally on the nervous system to suppress appetite and food intake [36]. It is also suggested that chronic administration of very high doses of GIP analogs may lead to GIPR desensitization, ultimately causing these substances to affect the receptors in a manner similar to GIP antagonists [61]. 

The action of GIP and GLP-1 may depend on Dipeptidyl peptidase-4 (DPP4) [64]. DPP4 is an enzyme responsible for the degradation and inactivation of these hormones. The secretion of the enzyme in the human body is still not sufficiently studied. However, most current analyses indicate that its primary source is adipose tissue, particularly in cases of increased adipose tissue and the development of obesity. Animal studies have shown that reduced levels of DPP4 are associated with decreased food and water intake, as well as increased energy expenditure, ultimately leading to weight loss. This effect is probably linked to decreased inactivation of gastrointestinal hormones, resulting in greater availability in the central nervous system and peripheral tissues. Additionally, some in vitro and animal studies have noted that elevated DPP4 production is associated with an unfavorable lipid profile. However, the precise mechanisms of action have not yet been fully explored and analyzed. In summary, DPP4 is an adipokine that significantly affects adipose tissue function as well as insulin secretion and tissue insulin sensitivity by influencing the activity of GIP and GLP-1. Despite numerous studies analyzing this enzyme, the clinical effects of its action remain unclear [64].

In summary, to best determine the role of gastrointestinal hormones in obesity therapy, it is essential to conduct more research to evaluate the optimal doses of these hormones’ agonists or antagonists. An important component of future research is to investigate all potential sites of hormone action, given that both central nervous system effects and impacts on adipose tissue and insulin sensitivity are significant for therapy. Future studies are essential, as both antagonists and agonists may prove useful in the treatment of obesity.

## 5. Conclusions

Ghrelin is a hunger hormone that contributes to increased accumulation of adipose tissue, particularly visceral fat.

Elevated levels of GIP may contribute to the accumulation of visceral fat, although the precise mechanisms of the hormone’s action are not fully understood.

The use of GIP receptor agonists, particularly in combination with GLP-1 receptor agonists, may positively affect body weight reduction, and glucose and lipid metabolism.

The use of GLP-1 analogs contributes to the reduction in adipose tissue through actions on the central nervous system as well as directly on adipose tissue.

Administration of PYY may stimulate weight loss by suppressing appetite and increasing the use of adipose tissue lipids as an energy source, additionally reducing systemic inflammation.

CCK may positively influence lipid metabolism in adipose tissue, reduce systemic inflammation and decrease insulin resistance.

Further research is needed to assess the interactive effects of hormones on adipose tissue.

The use of appropriate agonists or antagonists of gastrointestinal hormones may be a promising approach for treating metabolic syndrome and its complications.

## Figures and Tables

**Table 1:** Summary of the results of reviewed studies.

Hormone	Study	Description
Ghrelin	Theander-Carrillo et al., 2006 [25]	Animal study: Centrally administered exogenous ghrelin contributed to increased lipid storage and decreased metabolism.
	Sangiao-Alvarellos et al., 2009 [26]	Animal study: Centrally administered exogenous ghrelin increased the activity of enzymes responsible for lipid synthesis and decreased the activity of enzymes responsible for lipid oxidation.
	Davies et al., 2009 [27]	Animal study: Exogenous active ghrelin increased visceral fat content and decreased the activity of enzymes responsible for lipid oxidation.
	Abizaid et al., 2012 [28] Nogueiras 2021 [23]	Review: Ghrelin reduced the utilization of lipids as an energy source, increased fat intake and, consequently, the energy value of the diet, limited basal metabolic rate and thermogenesis, decreased spontaneous physical activity.
GIP	Nakamura et al., 2018 [37]	Animal study: The use of GIP receptor antagonists contributed to reduced lipid uptake and improved lipid metabolism, resulting in decreased weight gain.
	Sakane et al., 2019 [38]	Men with overweight or obesity undergoing a 2-week observation following specific dietary recommendations: In the group that reduced fat intake, there was a decrease in GIP secretion, which contributed to lower LDL cholesterol levels, reduced glycated hemoglobin, improved HOMA-IR index values and decreased visceral fat.
	Seino et al., 2022 [39]	Review: GIP contributed to increased body weight in individuals on a high-fat diet and enhanced lipid accumulation in adipose tissue.
	Kagdi et al., 2024 [40]	Review of studies: Both GIP receptor agonists and antagonists can positively influence adipose tissue function, and their effects may depend on other hormones, tissue location and physiological state.
	Regmi et al., 2024 [41]	Study on human adipocyte models and mouse models: Long-acting GIP receptor agonists differentially regulated adipose tissue functions both in a fasting state and postprandially. They worked with insulin to improve glucose and lipid clearance postprandially and increased lipid release during fasting with low insulin levels.
GLP-1	Kadouh et al., 2020 [48]	Individuals with overweight or obesity: Administration of liraglutide led to greater weight loss without loss of lean body mass. Mechanism of action: delayed gastric emptying, reduced appetite, effect on adipose tissue; however, the exact mechanism is unknown.
	Wegeberg et al., 2021 [49]	Individuals with type 2 diabetes and polyneuropathy: Administration of liraglutide and performance of adipose tissue biopsy. The GLP-1 analog led to reduced body weight without changes in the size of subcutaneous adipocytes, pericellular fibrosis or macrophage infiltration. There are no data on whether the GLP-1 analogue affected the composition of SAT as no changes in the levels of free fatty acids, CD163, leptin, or adiponectin were observed. GLP-1 had a more pronounced effect on reducing visceral fat compared to subcutaneous fat, but further detailed studies are needed.
	Liao et al., 2023 [50]	Meta-analysis: GLP-1 agonists affected the reduction in visceral fat and hepatic fat through their impact on the central nervous system and appetite suppression as well as by acting on GLP-1 receptors in adipose tissue and decreasing the formation of white adipose tissue and hepatic fat. This effect was demonstrated in both healthy individuals and those with non-alcoholic fatty liver disease and type 2 diabetes.
PYY	Adams et al., 2006 [54]	Animal study: Infusion of exogenous PYY contributed to reduced body weight and appetite, decreased fat mass without changes in lean body mass and increased fatty acid oxidation.
	van den Hoek et al., 2007 [56]	Animal study: Infusion of exogenous PYY decreased the respiratory quotient, thereby increasing fatty acid oxidation, reducing glucose utilization as an energy source, and enhancing fatty acid utilization. It also increased glucose uptake in adipose tissue.
	Abdel-Hamid et al., 2019 [53]	Animal study: Infusion of exogenous PYY in diabetic animals led to reduced food intake, body weight and visceral fat content. Additionally, it decreased mRNA expression of NF-kappaB in adipose tissue, which reduced IL-6 production and limited systemic inflammation.
Cholecystokinin	Plaza et al., 2018 [58]	Animal study: Infusion of CCK reduced expression of the protein ANGPTL-4, resulting in increased lipoprotein lipase activity in both SAT and VAT.
	Plaza et al., 2019 [59]	Animal study: Infusion of CCK, through its action on CCK2 receptors, increased the production and concentration of adiponectin. This effect was insulin-dependent.
	Plaza et al., 2022 [60]	Animal study: Infusion of CCK increased the expression of AQP7 in white adipose tissue, contributing to elevated glycerol levels in the blood. There was no effect on the rate of lipolysis.

## Abbreviation

OECD	Organization for Economic Co-operation and Development
G20	Group 20
EU28	European Union
WAT	white adipose tissue
BeAT	beige adipose tissue
BAT	brown adipose tissue
VAT	visceral white adipose tissue
SAT	subcutaneous white adipose tissue
IL-6	Interleukin 6
GLP-1	glucagon-like peptide-1
PYY	peptide YY
CCK	cholecystokinin
GOAT	ghrelin O-acyltransferase
GIP	gastric inhibitory polypeptide
BMI	body mass index
AQP7	aquaporin 7
DPP4	Dipeptidyl peptidase-4
